# FISH-Based Markers Enable Identification of Chromosomes Derived From Tetraploid *Thinopyrum elongatum* in Hybrid Lines

**DOI:** 10.3389/fpls.2018.00526

**Published:** 2018-05-01

**Authors:** Daiyan Li, Tinghui Li, Yanli Wu, Xiaohui Zhang, Wei Zhu, Yi Wang, Jian Zeng, Lili Xu, Xing Fan, Lina Sha, Haiqin Zhang, Yonghong Zhou, Houyang Kang

**Affiliations:** ^1^Triticeae Research Institute, Sichuan Agricultural University, Chengdu, China; ^2^College of Resources, Sichuan Agricultural University, Chengdu, China; ^3^Joint International Research Laboratory of Crop Resources and Genetic Improvement, Sichuan Agricultural University, Chengdu, China

**Keywords:** Fluorescence *in situ* hybridization karyotype, tetraploid *Thinopyrum elongatum*, repetitive sequences, molecular marker, hybrid derivatives

## Abstract

Tetraploid *Thinopyrum elongatum*, which has superior abiotic stress tolerance characteristics, and exhibits resistance to stripe rust, powdery mildew, and *Fusarium* head blight, is a wild relative of wheat and a promising source of novel genes for wheat improvement. Currently, a high-resolution Fluorescence *in situ* hybridization (FISH) karyotype of tetraploid *Th. elongatum* is not available. To develop chromosome-specific FISH-based markers, the hexaploid *Trititrigia* 8801 and two accessions of tetraploid *Th. elongatum* were characterized by different repetitive sequences probes. We found that all E-genome chromosomes could be unambiguously identified using a combination of pSc119.2, pTa535, pTa71, and pTa713 repeats, and the E-genome chromosomes of the wild accessions and the partial amphiploid failed to exhibit any significant variation in the probe hybridization patterns. To verify the validation of these markers, the chromosome constitution of eight wheat- *Th. elongatum* hybrid derivatives were analyzed. We revealed that these probes could quickly detect wheat and tetraploid *Th. elongatum* chromosomes in hybrid lines. K16-712-1-2 was a 1E (1D) chromosome substitution line, K16-681-4 was a 2E disomic chromosome addition line, K16-562-3 was a 3E, 4E (3D, 4D) chromosome substitution line, K15-1033-8-2 contained one 4E, two 5E, and one 4ES⋅1DL Robertsonian translocation chromosome, and four other lines carried monosomic 4E, 5E, 6E, and 7E chromosome, respectively. Furthermore, the E-genome specific molecular markers analysis corresponded perfectly with the FISH results. The developed FISH markers will facilitate rapid identification of tetraploid *Th. elongatum* chromosomes in wheat improvement programs and allow appropriate alien chromosome transfer.

## Introduction

As one of the most important cultivated cereals, the wheat (*Triticum aestivum* L., 2*n* = 6*x* = 42, AABBDD) grain production was forecast at 754.8 million tonnes in 2017, and ranked second only to maize in the world (FAO, 2017). However, the progress in wheat breeding is hampered by a relatively narrow range of genetic diversity (Dubcovsky and Dvorak, 2007). To solve this problem, it is good to develop cultivars with superior tolerance to biotic and abiotic stress. Wild relatives have a rich reservoir of useful alien genes that can be introduced into common wheat by distant hybridization to enrich the genetic diversity of wheat (Friebe et al., 1996; Mujeeb-Kazi et al., 2013; Ceoloni et al., 2017).

As a tertiary gene pool species for common wheat, *Thinopyrum elongatum* (syn. *Agropyron elongatum*, *Lophopyrum elongatum*) has always been a focus of wheat breeders. The taxon comprises a complex of polyploidy series based on the E-genome: diploid (2*n* = 2*x* = 14, EE), tetraploid (2*n* = 4*x* = 28, EEEE) and decaploid (2*n* = 10*x* = 70, EEEEEEStStStSt). It is commonly believed that the E-genome of the diploid is one of the basic genomes of *Th. elongatum* polyploids (Dvořák and Chen, 1984). Tetraploid *Th. elongatum* harbors genes that protect against many pathogens and adverse conditions including stripe rust, powdery mildew, FHB, smut, cold, drought, and high salinity (Dvořák et al., 1988; Jauhar and Peterson, 1996; Fedak, 1999). It also serves as an important wild gene pool to increase the genetic diversity of common wheat (Guo et al., 2015). To date, most introgressions from *Th. elongatum* into wheat have been produced using diploid and decaploid *Th. elongatum* (Sepsi et al., 2008; Ceoloni et al., 2017; Lou et al., 2017). Few attempts have been made to transfer the tetraploid *Th. elongatum* genes into wheat (Chen et al., 2013; Li et al., 2016; Dai et al., 2017a,b).

Fluorescence *in situ* hybridization (FISH) is a widely used cytogenetic tool in genomic studies of chromosome alterations (Alkhimova et al., 1999). The utility of FISH depends on the availability of suitable probes that provide chromosome-specific labeling patterns. FISH has become a new powerful tool for differentiating wheat chromosomes from those of its relatives (Sepsi et al., 2008; Zhuang et al., 2015). Afa family, pAs1, pSc119.2, pTa535, pTa713, pTa794, and pTa71 repetitive sequences are usually used as probes in FISH analysis to distinguish wheat A-, B-, and D-genome chromosomes and wheat wild species chromosomes (Rayburn and Gill, 1986; Fedak and Han, 2005; Molnár-Láng et al., 2010; Tang et al., 2014; Du et al., 2017; Kang et al., 2017). Linc et al. (2012) set up a detailed FISH karyotype of the E-genome of diploid *Th. elongatum* using probes pSc119.2, Afa family, and pTa71 probes, and revealed that significant intraspecific chromosome polymorphism in different accessions of diploid *Th. elongatum* with diverse geographical origin. Georgieva et al. (2011) detected significant signals on most chromosomes of *Th. intermedium* (2*n* = 6*x* = 42, EEJJStSt) with pSc119.2, Afa family, and pTa71 probes; only three pairs were not detected because they did not hybridize with any of the probes used. To date, knowledge of tetraploid *Th. elongatum* FISH patterns remains poor. A lack of standard karyotypes of tetraploid *Th. elongatum* has hampered its use for chromosome-mediated gene transfer into wheat.

The hexaploid *Trititrigia* 8801 (2*n* = 6*x* = 42, AABBEE), a genetically stable partial amphidiploid line, was produced by hybridization of *T. durum* (2*n* = 4*x* = 28, AABB) with tetraploid *Th. elongatum* (Guo et al., 2015). *Trititrigia* 8801 is commonly used as a bridge parent to transfer desirable genes from wild wheat species into cultivated wheat (Dai et al., 2017b). Currently, a standard FISH karyotype of tetraploid *Th. elongatum* has not been established. In addition, we cannot depend solely on the FISH karyotype of diploid *Th. elongatum* to identify the E chromosomes of tetraploid *Th. elongatum* due to polymorphism of the E-genome chromosomes from the two ploidy levels of *Th. elongatum*. Therefore, development of a FISH map for tetraploid *Th. elongatum* could help in alien chromosome identification in wheat- *Trititrigia* 8801 hybrid derivatives. Tetraploid *Th. elongatum* is an important genetic resource for wheat improvement and many wheat–tetraploid *Th. elongatum* alien chromosome introgression lines have been developed. The objectives of the present research were mainly to: (i) set up a FISH karyotype of the E-genome chromosomes of tetraploid *Th. elongatum* using different repetitive sequence probes; and (ii) accurately identify wheat–tetraploid *Th. elongatum* alien chromosome introgression lines and reveal genetic diversity among wheat cultivars by FISH-based markers.

## Materials and Methods

### Plant Materials

Materials employed in the current study are listed in **Table 1**. The hexaploid *Trititrigia* 8801 (2*n* = 6*x* = 42, AABBEE), which has the characteristics of cold, drought, and salt tolerance, and superior resistance to *Fusarium* head blight, rust, and powdery mildew, was kindly supplied by Dr. George Fedak (Eastern Cereal and Oilseed Research Center, Ottawa, Canada). Two accessions of tetraploid *Th. elongatum* (2*n* = 4*x* = 28, EEEE) with different origin were used for studying chromosome polymorphism: PI 531749 (Italy) and PI531750 (Greece), from the American National Plant Germplasm System (Pullman, WA, United States). The native wheat cultivars Shumai482 (SM482), Shumai921 (SM921), Shumai51 (SM51), Chuanmai104 (CM104), and Chuannong16 (CN16) are ideal recurrent parents for wheat breeding programs in southwestern China because they possess a comprehensive array of good agronomic characters, but most are susceptible to stripe rust pathogens prevalent in China. Eight derivative lines K16-712-1-2, K16-681-4, K16-562-3, K17-1089-1, K17-1084-3, K17-1065-5, K16-2521-1-2, and K15-1033-8-2 were obtained by hybridization of native wheat cultivars with *Trititrigia* 8801. For GISH analysis, the wheat cultivar ‘J-11’ (2*n* = 6*x* = 42, AABBDD) genome was used as blocking DNA, and the entire genomic DNA of tetraploid *Th. elongatum* PI531750 was used as a probe. Chinese Spring (CS, *T. aestivum* L., 2*n* = 6*x* = 42, AABBDD) was used as a control in molecular marker analysis.

**Table 1:** Plant materials used in this study.

Lines	Pedigree
PI531749, PI531750	Tetraploid *Thinopyrum elongatum* (2*n* = 4*x* = 28)
8801	*Triticum durum*- Tetraploid *Thinopyrum elongatum* amphidiploid (*Trititrigia*, AABBEE, 2*n* = 6*x* = 42)
SM482, SM921, SM51, CN16, CM104	Shumai482, Shumai921, Shumai51, Chuannong16, Chuanmai104
CS	Chinese Spring
J-11	Wheat cultivar J-11
K15-1033-8-2	8801/SM482//SM482 F_4_
K16-562-3	8801/SM482 F_5_
K16-681-4	8801/SM482//SM921 F_4_
K16-712-1-2	8801/SM482//SM921 F_5_
K16-2521-1-2	8801/SM482//SM51///CM104 F_4_
K17-1065-5	8801/SM482//SM921 F_5_
K17-1084-3	8801/CN16//SM51///SM51 F_3_
K17-1089-1	8801/SM482//SM482///SM482 F_4_

### GISH and FISH Analysis

Germinating seed root tips were collected and treated with nitrous oxide for 2 h and 90% acetic acid for 10 min, and the digested with pectinase and cellulose (Yakult Pharmaceutical Industry Co., Ltd., Tokyo, Japan), using the procedure of Komuro et al. (2013). Slides were prepared for GISH as previously described by Han et al. (2006). Total genomic DNA of tetraploid *Th. elongatum* PI531750 and J-11 was isolated using the cetyltrimethylammonium bromide (CTAB) method (Allen et al., 2006). The tetraploid *Th. elongatum* genomic DNA was labeled, as a probe, with fluorescein-12-dUTP using the nick translation mix (Thermo Fisher Scientific, Eugene, OR, United States). J-11 DNA was used for blocking, and as probe DNA and blocking agent DNA in the proportion of 1:150. GISH hybridization and detection were performed following the method of Han et al. (2009). A total volume of 10 μL of hybridization mixture containing 10 ng/μL labeled probe DNA, blocking DNA and buffer (2 × SSC and 1 × TE), was loaded per slide, denatured by heating at 85°C for 5 min, incubated for 8 h at 37°C, and washed in 2 × SSC. Chromosomes were counterstained with DAPI (4-6-diamino-2-phenylindole) solution (Vector Laboratories, Inc., Burlingame, CA, United States). Photomicrographs of GISH chromosomes were taken with an Olympus BX-51 (Olympus Corporation, Tokyo, Japan) microscope equipped with a DP-70 CCD camera and all images were processed with Photoshop CS 5.0 (Adobe Systems Incorporated, San Jose, CA, United States).

After the GISH analysis, the slides were washed with 2 × SSC and used for multicolor FISH. Synthetic oligonucleotide probes, including Oligo-pSc119.2, Oligo-pTa535, Oligo-pTa71, pAs1 (Tang et al., 2014), Oligo-pTa713 (Komuro et al., 2013), Oligo-pTa794 (Gerlach and Dyer, 1980), (ACT)_5_, (CTT)_5_, and Afa family (TAM 5′-GTTGAAACTTGGCATGGTATCATAATTTCACCCACATAGCATGT GCTAAAAAGTTGAGAGGGTTAC), were designed according to Nagaki et al. (1995). All the probes were synthesized by TSINGKE (Chengdu, China). The FISH procedure was also performed according to Han et al. (2006), with slight modifications. The probe mixture (0.35 μL of each probe in 2 × SSC and 1 × TE buffer, pH 7.0, total volume = 10 μL) was dropped on a slide, covered with a coverslip, stored in a moist box at 37°C for 2 h and washed in 2 × SSC at room temperature. The procedures for detection and visualization of FISH signals were the same as in the aforementioned GISH protocol.

### Molecular Marker Analysis

Seven *Th. elongatum* 1-7E specific markers were used to characterize the chromatin in the eight wheat-*Th. elongatum* introgression lines (all primer sequences are shown in **Table 2**). *Trititrigia* 8801 and tetraploid *Th. elongatum* PI531750 were used as positive controls, while CS, SM482, SM921, SM51, CM104, and CN16 were used as negative controls. The total volume of the amplification reaction system was 25 μL, including 1.0 μL of template DNA (100 ng/μL), 12.5 μL of 2 × Taq Master Mix for PAGE (Dye plus), 1.0 μL of 10 μM of each of the two primers, and 9.5 μL ddH_2_O. The PCR reaction procedure involved pre-denaturing at 94°C for 5 min; 35 cycles of 94°C for 45 s, 55-60°C for 1 min, and 72°C for 30 s; a final extension of 10 min at 72°C; and holding at 4°C. The PCR amplification products were detected by 3% agarose gel electrophoresis and visualized with a gel imaging system (Bio-Rad Laboratories, lnc., United States).

**Table 2:** Sequences of E-genome chromosomes specific markers.

Marker	Primer sequence (5′–3′)	Annealing temperature (°C)	Amplified chromosomes	Reference
M1E_No.21	F: CATCATATAAGAATGCAGGA R: TCAACAGCACGCCAAC	55	1E	Dai et al., 2017b
SLAF 2E-22	F: TTTTGATGGCGACTTTAGG R: CTTGGTTCCTGACCCTTT	55	2E	Dai et al., 2017b
SLAF 3E-6	F:GTCTACTTTAGCACCTCACTCA R: AAATTCGTCCGGTTGTT	60	3E	Dai et al., 2017b
P4E-1	F:TGTTGCCAGTGGAGGAAG R:TTGAACTACTGAACCGAAT	55	4E	Chen et al., 2015
P5E-1	F:ACAGTGAGCCCGAAGGAA R:AACACCAGCGGACAAAGC	60	5E	Chen et al., 2015
SLAF 6E-15	F: AGTAATTGGAGACCCAGGCG R: CAAATCAAACGACCATCACA	60	6E	Dai et al., 2017b
M7E_No.53	F:GTCAAGAGTTGGCTTTATTC R:ATTTGCTAATTCTCGTCATA	60	7E	Chen et al., 2013

## Results

### Characterizing Karyotype of Tetraploid *Th. elongatum* E-Genome Chromosomes

**FIGURE 1:**
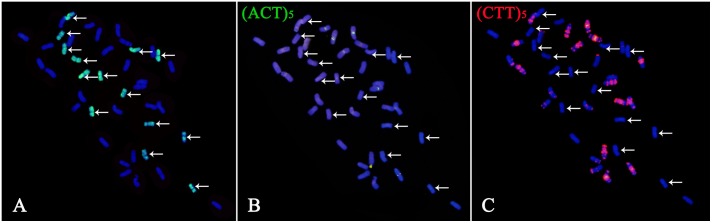
Karyotyping of mitotic metaphase chromosomes of *Trititrigia* 8801 by sequential GISH-FISH. Arrows indicate E-genome chromosomes. **(A)** Tetraploid *Thinopyrum elongatum* genomic DNA was used as a probe for GISH (green). **(B,C)** (ACT)_5_ and (CTT)_5_ probe signals were pseudo-colored as green and red in the FISH patterns, respectively. The probes (ACT)_5_ and (CTT)_5_ failed to show any signals on the E-genome chromosomes.

**FIGURE 2:**
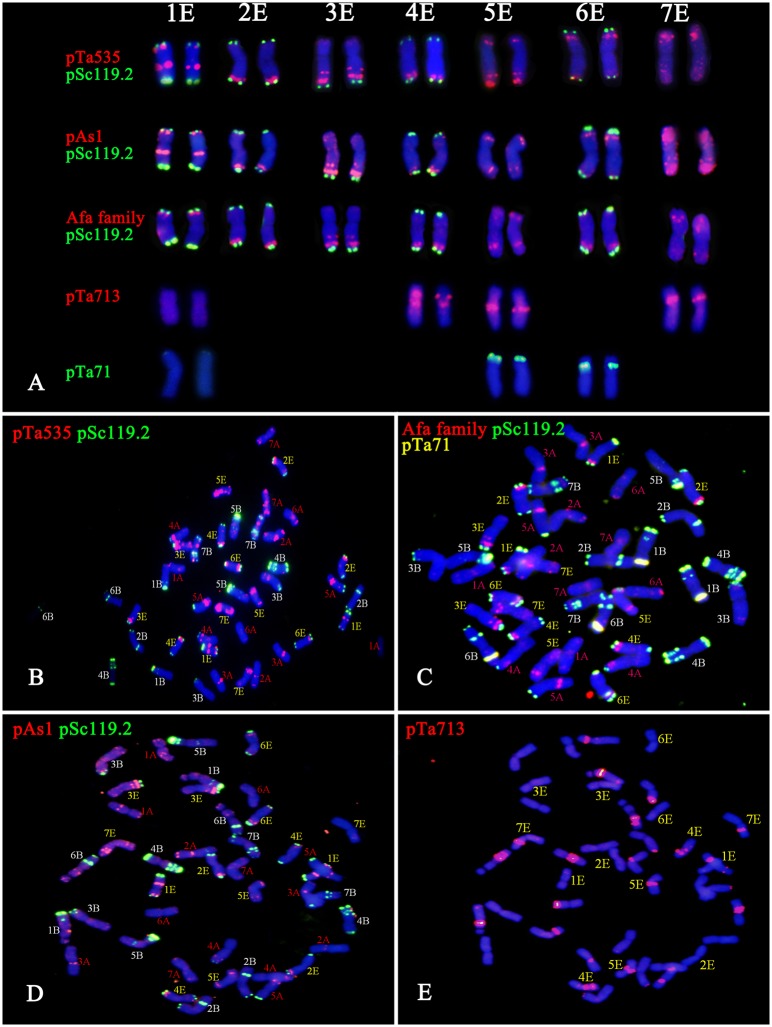
FISH patterns of oligo probes in *Trititrigia* 8801. **(A)** FISH patterns of the 1E-7E chromosomes using pSc119.2 (green), pTa535 (red), Afa family (red), pAs1 (red), pTa713 (red), and pTa71 (green). **(B)** FISH identification of 8801 using pTa535 (red) and pSc119.2 (green). **(C)** FISH identification of 8801 using Afa family (red), pSc119.2 (green), and pTa71 (yellow). **(D)** FISH identification of 8801 using pAs1 (red) and pSc119.2 (green). **(E)** FISH identification of 8801 using pTa713 (red).

**FIGURE 3:**
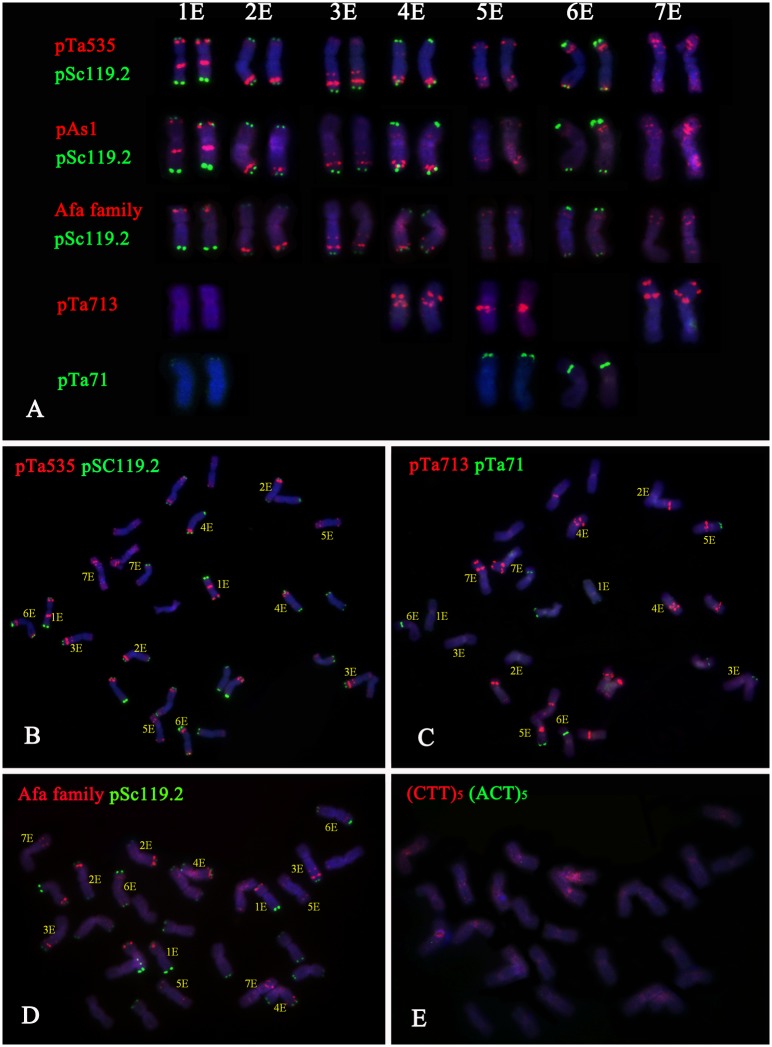
FISH patterns of the tetraploid *Th. elongatum*. **(A)** FISH patterns of the 1E-7E chromosomes using pSc119.2 (green), pTa535 (red), Afa family (red), pAs1 (red), pTa713 (red), and pTa71 (green). **(B)** FISH identification of PI531750 using pTa535 (red) and pSc119.2 (green). **(C)** FISH identification of PI531750 using pTa713 (red) and pTa71 (green). **(D)** FISH identification of PI531749 using Afa family (red) and pSc119.2 (green). **(E)** FISH identification of PI531749 using (ACT)_5_ and (CTT)_5_. The probes (ACT)_5_ and (CTT)_5_ failed to show any signals on the E-genome chromosomes.

The GISH results showed that *Trititrigia* 8801 carried 14 E-genome chromosomes (**Figure 1A**). The GISH signals were then washed away and the same slide was used for FISH. After screening and combining FISH probes including (ACT)_5_, (CTT)_5_, pSc119.2, pAs1, pTa535, Afa family, pTa71, pTa713, and pTa794, the (ACT)_5_ and (CTT)_5_ microsatellites failed to show any signals on any E-genome chromosomes (**Figures 1B,C**). The seven pairs of E-genome chromosomes of tetraploid *Th. elongatum* could be accurately identified in *Trititrigia* 8801 using the repeat combination pSc119.2, pTa535, pTa71, and pTa713; additionally, the probe pTa535 could also be replaced by pAs1 or Afa family (**Table 3** and **Figure 2**). Two accessions of tetraploid *Th. elongatum* failed to show significant intraspecific chromosome polymorphism after FISH using repetitive clones (**Figure 3**). The probe hybridization patterns of the E-genome chromosomes were similar between the wild accessions and *Trititrigia* 8801, except for 7E chromosomes using pTa713 probe (**Figures 2**, **3**).

**Table 3:** FISH identification of tetraploid *Th. elongatum.*

Probes	E-genome chromosomes							
	1E	2E	3E	4E	5E	6E	7E
pSc119.2	+	+	+	+	-	+	-
pTa535	+	+	+	+	+	+	+
pAs1	+	+	+	+	+	+	+
Afa family	+	+	+	+	+	+	+
pTa713	+	-	-	+	+	-	+
pTa71	+	-	-	-	+	+	-
pTa794	+	-	-	-	-	-	-
(ACT)_5_	-	-	-	-	-	-	-
(CTT)_5_	-	-	-	-	-	-	-

*“+” positive signals; “-” no signals.*

Chromosome 1E carried strong terminal pSc119.2 signals on both arms, and strong pTa535 signals were found on the subterminal region of the short arm and near the centromere region of the long arm. The pAs1 signals on chromosome 1E were similar to those of pTa535, but differed from those of the Afa family probe, which had subterminal signals on the short arm. Faint pTa71 and pTa713 hybridization sites were detected on the short arm and centromere region of 1E chromosome, respectively. Chromosome 2E was characterized by strong terminal signals of pSc119.2 on both arms and subterminal pTa535 signals on the long arm. Clear terminal pSc119.2 signals and two subterminal pTa535 signals were detected on the long arm of chromosome 3E, and the short arm produced faint telomeric pTa535 signals. Chromosome 4E had similar pSc119.2 and pTa535 signals compared with chromosome 2E, but showed faint pTa535 signals subterminally on the short arm. However, strong pTa713 signals were detected near the centromere region of chromosome 4E, which distinguished it from chromosome 2E. We failed to detect any pSc119.2 signals on chromosome 5E, but pTa535 signals were found on the terminal region of the short arm and the terminal-subterminal region of the long arm. The probe pTa713 gave strong signals near the centromere on the long arm of chromosome 5E. Chromosome 6E was identified by strong terminal signals of pSc119.2 and subterminal signals of pTa535 on both arms. Notably, chromosomes 1E, 5E, and 6E carried different 45S rDNA signals. Chromosome 7E lacked any specific pSc119.2 signals, while strong pTa535 signals were located in terminal-subterminal positions and strong pTa713 signals were found near the centromere on the short arm. In addition, the pAs1 and Afa family signals were similar to those of pTa535 on the E chromosomes of groups 2, 3, 4, 5, 6, and 7 (**Figure 2**).

### Alien Chromosome Identification of Hybrid Progenies Using Efficient Oligo Probes

**FIGURE 4:**
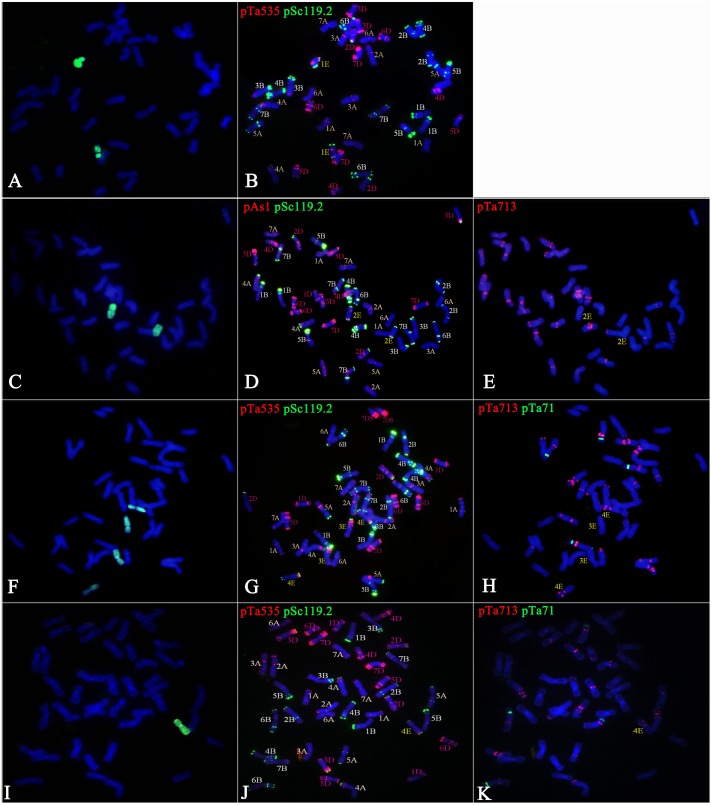
Chromosome identification of hybrid progenies. Images on the right of each panel show FISH results using pSc119.2 (green), pTa535 (red), pAs1 (red), pTa713 (red), and pTa71 (green), and those on the left show corresponding GISH patterns. Chromosomes in blue and green are wheat and *Th. elongatum* chromosomes in the GISH patterns, respectively. **(A,B)** Line K16-712-1-2 had a complete set of 28 A/B chromosomes, 12 D (2D-6D) chromosomes, and a pair of 1E chromosomes. **(C–E)** Line K16-681-4 had 13 A (lacking one 3A), 16 B (four 7B), 13 D (lacking one 1D), and one pair of 2E chromosomes. **(F–H)** Line K16-562-3 had all 28 A/B chromosomes, 10 D (1D, 2D, 5D, 6D, and 7D) chromosomes, and a pair each of 3E and 4E chromosomes. **(I–K)** Line K17-1089-1 had a complete set of 42 A/B/D chromosomes and one 4E chromosomes.

**FIGURE 5:**
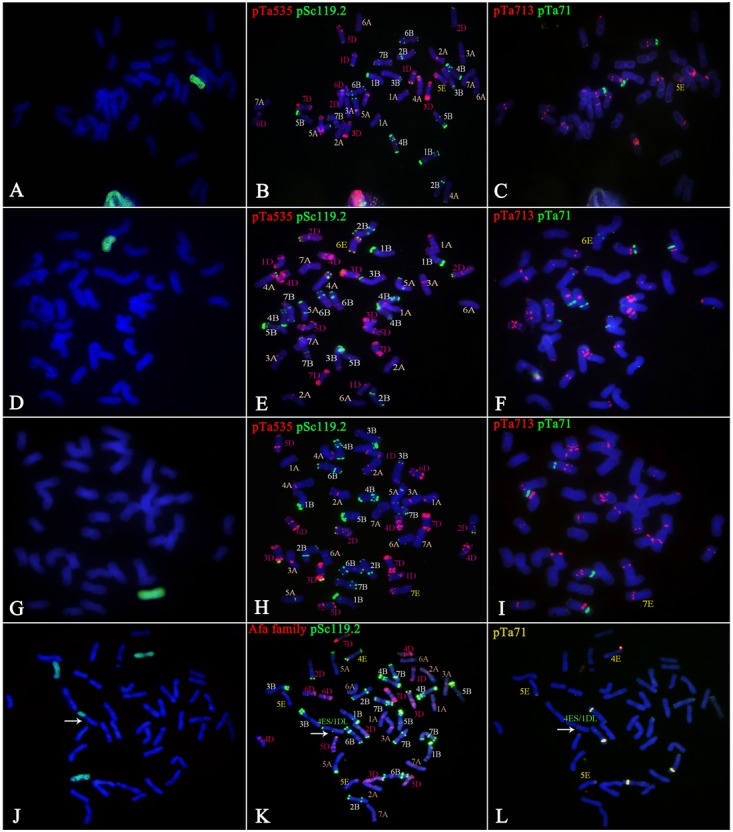
Chromosome identification of the hybrid progenies. Images on the right of each panel show FISH results using pSc119.2 (green), pTa535 (red), pAs1 (red), pTa713 (red), and pTa71 (green), and those on the left show corresponding GISH patterns. Chromosomes in blue and green are wheat and *Th. elongatum* chromosomes in the GISH patterns, respectively. **(A–C)** Line K17-1084-3 had a complete set of 28 A/B chromosomes, 12 D (lacking one 4D and 5D) chromosomes, and one 5E chromosomes. **(D–F)** Line K17-1065-5 had 14 A, 15 B (plus one 4B), 12 D (1D-5D, 7D), and one 6E chromosomes. **(G–I)** Line K16-2521-1-2 had a complete set of wheat chromosomes and one 7E chromosomes. **(J–L)** K15-1033-8-2 had 12A (1A-3A, 5A-7A), 16 B (four 7B), 13 D (lacking one 1D), 3 E (one 4E and two 5E), and one 4ES1DL translocation chromosomes (arrow).

GISH analysis was used to detect E-genome chromosomes in eight wheat-*Trititrigia* 8801 hybrid derivatives. Then, according to the FISH pattern developed for the E-genome chromosomes of tetraploid *Th. elongatum*, the chromosome constitutions of these lines were accurately identified (**Table 4** and **Figures 4**, **5**). The FISH analysis showed that the line K16-712-1-2 was a 1E (1D) chromosome substitution line, containing 14 A-genome (1A-7A), 14 B-genome (1B-7B), 12 D-genome (2D-7D), and one pair of 1E chromosomes (**Figures 4A,B**). The probe signals indicated that the line K16-681-4 was a disomic chromosome addition line with a pair of 2E chromosomes (2*n* = 44) (**Figures 4C–E**). Line K16-562-3 was a 3E, 4E (3D, 4D) chromosome substitution line that contained 14 A-genome, 14 B-genome, 10 D-genome, and two pairs of E-genome chromosomes (**Figures 4F–H**). Line K17-1089-1 (2*n* = 43) consisted of one 4E chromosome and complete sets of A-, B-, and D-genome chromosomes (**Figures 4I–K**). There were 40 wheat chromosomes and one 5E chromosome in K17-1084-3 (**Figures 5A–C**). Line K17-1065-5 (2*n* = 42) carried all A-genome and B-genome (plus one 4B) chromosomes, and 12 D-genome (lacking two 6D) plus one 6E chromosomes (**Figures 5D–F**). Line K16-2521-1-2 was a monosomic addition line that contained the complete sets of A-, B-, and D-genome chromosomes and one 7E chromosomes (**Figures 5G–I**). Additionally, line K15-1033-8-2 contained 41 chromosomes of common wheat, three E-genome (one 4E and two 5E) chromosomes, and one wheat-*Th. elongatum* Robertsonian translocation chromosome (4ES⋅1DL) (**Figures 5J–L**).

**Table 4:** Chromosome constitution of eight hybrid progenies.

Line	Chromosome constitution
K16-712-1-2	2*n* = 42 = 40W + II 1E
K16-681-4	2*n* = 44 = 42W + II 2E
K16-562-3	2*n* = 42 = 38W + II 3E + II 4E
K17-1089-1	2*n* = 43 = 42W + I 4E
K17-1084-3	2*n* = 41 = 40W + I 5E
K17-1065-5	2*n* = 42 = 41W + I 6E
K16-2521-1-2	2*n* = 43 = 42W + I 7E
K15-1033-8-2	2*n* = 45 = 41W + I 4E + II 5E + I 4ES/1DL

*E, oxE-genome chromosomes of Th. elongatum; W A-, B-, and oxD-genome chromosomes of wheat.*

### Molecular Marker Analysis

**FIGURE 6:**
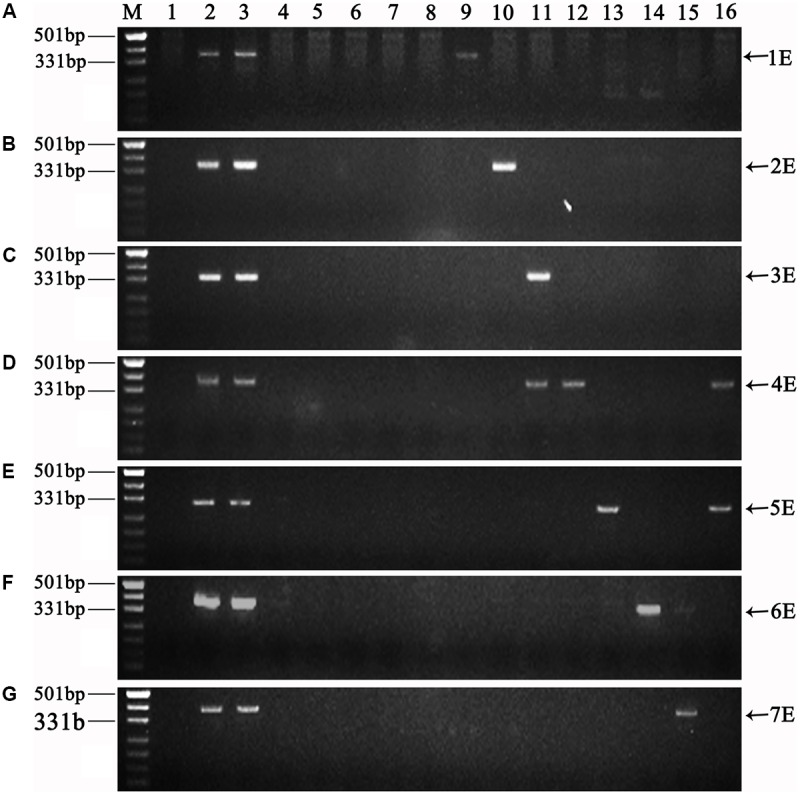
Amplification patterns of E-genome specific molecular markers. **(A-G)** Specific molecular markers of chromosomes 1E to 7E. Lanes: M Marker (DL500); 1 CS; 2 Tetraploid *Th. elongatum* PI531750; 3 8801; 4 SM482; 5 SM921; 6 SM51; 7 CM104; 8 CN16; 9 K16-712-1-2; 10 K16-681-4; 11 K16-562-3; 12 K17-1089-1; 13 K17-1084-3; 14 K17-1065-5; 15 K16-2521-1-2; 16 K15-1033-8-2. *Arrows* show the diagnostic amplification products of tetraploid *Th. elongatum* 1E-7E chromosomes.

The chromosome constitutions of the eight hybrid progenies were verified using E-genome specific molecular markers. The PCR results are shown in **Figure 6**. As expected, amplification products with the 1E, 2E, 4E, 5E, 6E, and 7E specific markers were observed in lines K16-712-1-2, K16-681-4, K17-1089-1, K17-1084-3, K17-1065-5, K16-2521-1-2, respectively, and the parents *Trititrigia* 8801 and tetraploid *Th. elongatum* PI531750. Similarly, line K16-562-3 showed bands of 3E and 4E chromosomes, and 4E and 5E chromosomes were detected in line K15-1033-8-2. Thus, the PCR results for the chromosome constitutions of the hybrid progenies were consistent with the FISH analysis.

## Discussion

The ultimate objective of this study was to set up an E-genome specific FISH karyotype of tetraploid *Th. elongatum* using different probes, and determine the chromosome constitutions of wheat- tetraploid *Th. elongatum* progenies. A FISH pattern for tetraploid *Th. elongatum* was produced using the probes pSc119.2, pTa535, pTa713, and pTa71, enabling the exact FISH characterization of the E-genome chromosomes in wheat-tetraploid *Th. elongatum* hybrids.

Many studies have shown that repetitive DNA sequences are major components of plant genomes. For example, they can account up to 80% of the genome size in common wheat and barley (Flavell et al., 1974; Mascher et al., 2017; Wang et al., 2017). Using repetitive sequences as probes, FISH has proved to be a valuable tool for accurate identification of individual chromosomes within a species from the distribution of hybridization sites (Rayburn and Gill, 1986) or illustrating the phylogenetics of polyploidy species by analyzing the intraspecific and interspecific divergence (Taketa et al., 2000; Arzani et al., 2005; Zhao et al., 2018). The microsatellites (ACG)n, (ACT)n, (GAA)n, and complementary repeat (CTT)n have been used to identify the chromosomes of common wheat, rye, and *Aegilops* species (U-, M-, S-, and D-genomes) (Molnár et al., 2011; Schneider and Molnár-Láng, 2012; Badaeva et al., 2015; Zhao et al., 2016). However, the sequences (ACT)_5_ and (CTT)_5_ failed to show any signals on the E-genome chromosomes in the present study, which is consistent with previous studies (Sepsi et al., 2008; Linc et al., 2012). The probe pTa535 from wheat repetitive sequences enables the discrimination of A- and D-genome chromosomes (Komuro et al., 2013). In this study, pTa535 produced characteristic patterns on all chromosomes of tetraploid *Th. elongatum*, and facilitated the detection of the E-genome chromosomes in the wheat background. The pSc119.2 probes from rye repetitive sequences was used to identify the B-genome and 4A, 5A, 2D, 3D, and 4D chromosomes of wheat and R-genome chromosomes of rye (Cuadrado et al., 1997; Tang et al., 2014). pSc119.2 has also been successfully used to detect some *Thinopyrum* chromosomes, including those of *Th. intermedium*, *Th. ponticum*, *Th. bessarabicum*, and diploid *Th. elongatum* (Lapitan et al., 1987; Charette and Andre, 2003; Georgieva et al., 2011; Linc et al., 2012; Du et al., 2017). Previous studies on Triticeae have shown that, in most species, combinations of pSc119.2 and other FISH probes do not permit the complete identification of all chromosomes. In our results, five chromosomes (1E, 2E, 3E, 4E, and 6E) hybridized with pSc119.2, which made them distinguishable from the pattern of the well-known CS chromosomes, but there was no difference between chromosomes 2E and 4E. However, using the probe pTa713, clear signals were visible in the pericentromeric regions of the 1E, 4E, 5E, and 7E chromosomes. Therefore, we concluded that FISH with pSc119.2 and pTa713 enabled the identification of all tetraploid *Th. elongatum* chromosomes. pTa794 is a 420 bp-long sequence of wheat encoding the 5S rRNA gene (Gerlach and Dyer, 1980). The pTa71 sequence is a clone from common wheat with a size of 9 kb and encodes 18S, 5.8S, and 26S rRNA genes (Gerlach and Bedbrook, 1979). The 45S rDNA sites preferentially occur in the chromosomes of homoeologous groups 1, 5, and 6 (Badaeva et al., 1996). The activity of the 45S rDNA genes is usually linked with NORs and secondary constrictions in the Triticeae tribe. Previous results using molecular markers indicated that the chromosome 1ES of diploid *Th. elongatum* carried 5S rDNA sites, and the 5E and 6E chromosomes were characterized by two clear 45S rDNA sites (Dvořák et al., 1989; Fominaya et al., 1997). In Linc (2012), pTa71 failed to show any signals on the diploid *Th. elongatum* 6ES chromosome by FISH, but showed distinct subterminal 45S rDNA sites on chromosome 5ES. However, the present results showed that 5S rDNA sites were located on the 1ES chromosome of tetraploid *Th. elongatum* and 45S rDNA sites were simultaneously located on the short arms of the 1E, 5E, and 6E chromosomes. Polyploid speciation usually and rapidly cause genomic changes (Adams and Wendel, 2005). The 1E, 5E, and 6E chromosomes in this study showed different 45S rDNA sites compared with the diploid *Th. elongatum*, which can probably be attributed to evolutionary and environmental causes. Hence, using specific probes, the FISH karyotype of tetraploid *Th. elongatum* and the identities of eight wheat–tetraploid *Th. elongatum* alien chromosome introgression lines were characterized in a simple, fast, and accurate way. Our results demonstrate that FISH karyotyping can facilitate the use of tetraploid *Th. elongatum* in alien gene introgression into wheat.

Fluorescence *in situ* hybridization karyotype studies have been reported for various species in the Triticeae, such as *T. aestivum*, *Secale cereale*, *Ae. variabilis*, and *Agropyron cristatum* (Badaeva et al., 2004; Tang et al., 2014; Zhao et al., 2016; Linc et al., 2017). In the genus *Thinopyrum*, karyotypes for diploid *Th. elongatum* (Linc et al., 2012), *Th. bessarabicum* (Du et al., 2017), and *Th. intermedium* (Georgieva et al., 2011) have been completed and used to identify alien chromosomes in wheat introgression lines. In our study, we discriminated the chromosome constitutions of eight wheat-tetraploid *Th. elongatum* hybrid progenies by combining FISH karyotyping, GISH, and E-genome specific molecular markers. These progenies included a 1E (1D) substitution line, a 2E disomic addition line, a 3E, 4E (3D, 4D) disomic substitution line, and four lines with monosomic 4E, 5E, 6E, and 7E chromosomes, respectively. Furthermore, one line, K15-1033-8-2, contained a Robertsonian translocation chromosome, which was successfully distinguished. The results suggested that E-genome chromosomes of tetraploid *Th. elongatum* can be quickly and accurately identified by FISH karyotyping in the background of common wheat. Because some lines with monosomic alien chromosomes are cytogenetically unstable, the stable disomic substitution lines and translocation lines will be obtained by the selfing of the monosomic addition lines. These wheat-tetraploid *Th. elongatum* progeny lines will extend the genetic resources available for wheat breeding, and enrich the genetic diversity and improve the yield and quality of wheat. Further work is needed to evaluate the agronomic performance and disease resistance of these addition, substitution and translocation lines.

## Author Contributions

HK conceived and designed the research. DL, TL, YlW, and XZ conducted the experiments. WZ, YW, JZ, LX, XF, and LS participated in the preparation of the reagents and materials. HZ, YZ, and HK analyzed the data. DL and HK wrote the manuscript. All authors read and approved the manuscript.

## Conflict of Interest Statement

The authors declare that the research was conducted in the absence of any commercial or financial relationships that could be construed as a potential conflict of interest.
